# Virulence-related *Mycobacterium avium *subsp *hominissuis *MAV_2928 gene is associated with vacuole remodeling in macrophages

**DOI:** 10.1186/1471-2180-10-100

**Published:** 2010-04-01

**Authors:** Samradhni S Jha, Lia Danelishvili, Dirk Wagner, Jörg Maser, Yong-jun Li, Ivana Moric, Steven Vogt, Yoshitaka Yamazaki, Barry Lai, Luiz E Bermudez

**Affiliations:** 1 Department of Biomedical Sciences, College of Veterinary Medicine, Oregon State University, Corvallis OR 97331, USA; 2 Department of Internal Medicine II—Infectious Diseases, University of Freiburg, 79106 Freiburg, Germany; 3 Argonne National Laboratory, Argonne, Illinois, USA; 4 Geron Corporation, 230 Constitution Drive, Menlo Park, CA 94025, USA; 5 Department of Respiratory and Infectious Diseases, Shinshu University School of Medicine, 3-1-1 Asahi, Matsumoto, 390-8621, Japan; 6 Department of Microbiology, College of Science, Oregon State University, Corvallis OR 97331, USA

## Abstract

**Background:**

*Mycobacterium avium *subsp *hominissuis *(previously *Mycobacterium avium *subsp *avium*) is an environmental organism associated with opportunistic infections in humans. *Mycobacterium hominissuis *infects and replicates within mononuclear phagocytes. Previous study characterized an attenuated mutant in which the PPE gene (MAV_2928) homologous to Rv1787 was inactivated. This mutant, in contrast to the wild-type bacterium, was shown both to have impaired the ability to replicate within macrophages and to have prevented phagosome/lysosome fusion.

**Results:**

MAV_2928 gene is primarily upregulated upon phagocytosis. The transcriptional profile of macrophages infected with the wild-type bacterium and the mutant were examined using DNA microarray, which showed that the two bacteria interact uniquely with mononuclear phagocytes. Based on the results, it was hypothesized that the phagosome environment and vacuole membrane of the wild-type bacterium might differ from the mutant. Wild-type bacterium phagosomes expressed a number of proteins different from those infected with the mutant. Proteins on the phagosomes were confirmed by fluorescence microscopy and Western blot. The environment in the phagosome of macrophages infected with the mutant differed from the environment of vacuoles with *M. hominissuis *wild-type in the concentration of zinc, manganese, calcium and potassium.

**Conclusion:**

The results suggest that the MAV_2928 gene/operon might participate in the establishment of bacterial intracellular environment in macrophages.

## Background

*Mycobacterium avium *subsp *hominissuis *is an environmental organism commonly found in soil and water [1,2]. *Mycobacterium hominissuis *causes disseminated disease in immunocompromised people such as in AIDS patients, and disease in patients suffering from chronic pulmonary conditions [3]. The bacterium preferentially infects tissue macrophages and blood monocytes. Once inside a macrophage, the bacterium has been shown to inhibit the acidification of the phagosome and subsequently prevent the fusion between the phagosome and lysosome [4], which are key stages of phagocytes mechanisms of killing of intracellular microorganisms [5]. Similar to *Mycobacterium tuberculosis *[6], *Salmonella *[7] and *Leishmania *[8], *M. hominissuis *interferes with the endosome maturation process which precedes phagosome-lysosome fusion. The mechanisms that *M. hominissuis *uses to survive within macrophages have been an active area of research. Previous reports have shown that *M. hominissuis *has the ability to modulate the intracellular environment, remaining accessible to internalized transferrin and limiting the proteolytic activity, maintaining cathepsin D in an immature form [9]. Other studies, for example, Malik and colleagues, have suggested inhibition of calcium signaling by another pathogenic mycobacterium (*M. tuberculosis*) is responsible for the prevention of phagosome-lysosome fusion [10]. Li and colleagues [11], screening of *M. hominissuis *transposon mutant bank for clones with attenuated in human macrophages, identified a 2D6 mutant in which the transposon interrupted MAV_2928 a PPE gene (52% homologous to Rv1787 in *M. tuberculosis*). MAV_2928 is expressed primarily upon macrophage phagocytosis [11]. The 2D6 mutant was significantly attenuated in macrophages in comparison to the wild-type bacterium although both bacteria had comparable ability to enter the phagocytic cells. In addition, vacuoles containing the 2D6 mutant could not prevent the acidification and subsequent fusion with the lysosomes.

The PE, PPE, and PE-PGRS families of genes in mycobacteria are dispersed throughout the genomes of *M. tuberculosis*, *Mycobacterium bovis*, *M. hominissuis *and *Mycobacterium paratuberculosis*. It was previously assumed that *M. hominissuis *and *M. paratuberculosis *lack PE-PGRS family of proteins [12], but we have recently found PE-PGRS proteins in *M. hominissuis *(Li, Y and colleagues, in press). These families of proteins have been associated with virulence of mycobacteria [11,13,14], and some of the proteins have been identified on the bacterial surface [13]. The function of the majority of PPE proteins is unknown. Recently, work with *M. tuberculosis *has demonstrated that PPEs are associated with the RD1 operon and participate in the secretion of ESAT-6 and CFP-10, two proteins associated with *M. tuberculosis *virulence [15].

Early events during the infection are likely to influence the characteristics of the macrophage vacuole. MAV_2928 gene in *M. hominissuis*, homologue to *M. tuberculosis *Rv1787, is expressed upon entry in macrophages and, therefore, may participate in the establishment of the *M. hominissuis *environment within the phagocytic cell. Very little has been published on the proteins that make the bacterial vacuole. A study by Gagnon and colleagues [16] described the membrane proteins of latex bead vacuoles. Although some of the bacterial vacuole proteins have been determined, it is unknown how vacuoles recruit most of the proteins, and if bacterial vacuoles differ depending on the pathogen present within it. Previous studies have demonstrated that the intravacuolar environment is influenced by pathogens [6,17]. Whether this ability is related, at least in part, to changes in vacuole membrane is currently unknown. The intent of this research was to investigate whether the lack of a functional MAV_2928 would have any influence on the vacuole structure and intravacuolar environment.

## Results

### Differential gene induction in U937 cells after infection with MAC 109 and 2D6 attenuated mutant by DNA microarray

Because the MAV_2928, homologue to Rv1787, was shown to be upregulated upon initial contact between *M. avium *and macrophages, we decided to examine whether and how the macrophage transcription varies upon 2D6 mutant uptake compared to the gene expression triggered by the uptake of the wild-type bacterium. Tables 1 and 2 show the genes differentially regulated when comparing the wild-type bacterium and the 2D6 mutant. The genes induced in cells infected with wild-type bacteria, but not in cells infected with the 2D6 mutant, consisted mainly of those involved in intracellular signaling, such as LCK, PKIA, DGKA, DGKD, INPP1, APBA2 and PDE1C. A few other genes were involved in the metabolic pathways, such as GPD2 (involved in glycerol-3-phosphate metabolism) and CYP4F2 (involved in leukotriene metabolism). Additional genes that showed induction were PPM1G (cell cycle arrest), HIPK3 and RORC (inhibition of apoptosis), ITK (T-cell proliferation and differentiation), GRK4 (regulation of G-protein coupled receptor protein signaling), NFKB1 (transcriptional regulator) and others. The genes with decreased expression in wild-type but upregulated in 2D6 mutant included genes involved in signal transduction (BMX, CCR3, GPR17, GABBR1, GABBR2, YWHAZ, RAB7, RAB13, IFNA1, DGKZ and DGKG), apoptosis (BLK, GZMA), bacterial uptake (ITGB1, CR1), immune response (IL10RA, TNFRSF17, MS4A1, LCP2), metabolic pathways (DDOST, PLTP), and others, such as bacterial killing (cathepsin G), negative regulators of G-protein signaling (RGS12 and RGS13), potassium channel regulator (CHP), microtubule movement (TUBB, DCTN1, CETN2 and S100A11).

**Table 1:** Differential macrophage gene expression in *M. avium *109 and 2D6 mutant

Gene	Gene Bank ID	Name	Function	Fold induction (± SD)	p value <0.05
APBA2	AB014719	Amyloid beta (A4) precursor protein binding	Signal transduction	10.7 ± 2.3	Y
CYP4F2	U02388	Cytochrome P450	Inactivation & degradation of leukotriene B4	2.6 ± 0.9	Y
DGKA	AF064767	Diacylglycerol kinase alpha	Intracellular signaling	2.3 ± 0.4	Y
DGKD	D63479	Diacylglycerol kinase delta	Phosphatidylinositol signaling	6.7 ± 1.2	Y
DYNC1H1	AB002323	Cytosolic dyenin heavy chain	Microtubule reorganization	17.4 ± 3.1	Y
GPD2	NM_000408	Glycerol-3-phosphate dehydrogenase 2	Glycerol-3-phosphate metabolism	3.5 ± 0.4	Y
GRK4	NM_005307	G-protein coupled receptor kinase 4	Regulation of G-protein coupled receptor protein signaling	3.5 ± 0.6	Y
HIPK3	AF004849	Homeodomain interacting protein kinase 3	Inhibition of apoptosis	2.05 ± 0.3	Y
INPP1	NM_002194	Inositol polyphosphate-1-phosphatase	Phosphatidylinositol signaling	2.0 ± 0.4	Y
ITK	D13720	IL2-inducible T-cell kinase	T-cell proliferation & differentiation	2.4 ± 0.4	Y
LCK	M36881	Lymphocyte-specific protein tyrosine kinase	Intracellular signaling	3.5 ± 0.7	Y
NFKB1	M58603	Nuclear factor of kappa light polypeptide gene enhancer in B-cells 1	Transcriptional regulator	2.3 ± 0.4	Y
PDE1C	U40371	Calcium/calmodulin-dependant 3', 5'-cyclic nucleotide phosphodiesterase 1C	Signal transduction	17.4 ± 1.9	Y
PKIA	S76965	Protein kinase (cAmp-dependent) inhibitor alpha	Negative regulation of protein kinase A	2.0 ± 0.3	Y
PPM1G	Y13936	Serine/threonine protein phosphatase PP1-gamma 1 catalytic subunit	Negative regulator of cell stress response/cell cycle arrest	3.2 ± 0.5	Y
PTPN11	D13540	Protein tyrosine phosphatase	Intracellular signaling, cell migration	2.4 ± 0.2	Y
RGS3	AF006610	Regulator of G-protein signaling-3	Inhibition of G-protein mediated signal transduction	3.4 ± 0.3	Y
RORC	U16997	RAR-related orphan receptor C	Inhibition of Fas ligand and IL2 expression	3.1 ± 0.3	Y
ROR1	M97675	Receptor tyrosine kinase-like orphan receptor 1	Unknown	4.0 ± 0.4	Y

Complemented 2D6 mutant had similar results to the wild-type bacterium. Y = Yes; N = No

**Table 2:** Macrophage genes with decreased expression in *M. avium *109 but increased in 2D6 mutant 4 h post infection

Gene	Gene Bank ID	Name	Function	Fold induction (± SD)	p value <0.05
**AMBP**	X04494	**Alpha-1-microglobulin**	**Negative regulation of immune response/Protease inhibitor**	**4.2 ± 0.7**	**Y**
BLK	BC004473	B-lymphoid tyrosine kinase	Apoptosis	3.3 ± 0.3	Y
BMX	AF045459	BMX non-receptor tyrosine kinase	Intracellular signaling	18.6 ± 4.1	Y
CCR3	AF247361	Chemokine receptor 3	Signal transduction	4.1 ± 0.6	Y
CD53	BC040693	CD53 molecule	Growth regulation	4.1 ± 0.3	Y
CETN2	X72964	Centrin, EF-hand protein 2	Microtubule organization center	6.3 ± 0.9	Y
CHP	NP_009167	Calcium binding protein P22	Potassium channel regulator/Signal transduction	20.8 ± 3.5	Y
CR1	Y00816	Complement receptor 1	Bacterial uptake	4.3 ± 0.4	Y
CTSG	NM_001911	Cathepsin G	Bacterial killing	2.9 ± 0.2	Y
DCTN1	NM_004082	Dynactin 1	Lysosome and endosome movement	35.8 ± 8.0	Y
DDOST	D29643	Dolichyl-diphosphooligosaccharide-protein glycosyltransferase	N-linked glycosylation	3.3 ± 0.3	Y
DGKG	AF020945	Diacylglycerol kinase gamma	Intracellular signaling	5.3 ± 0.6	Y
DGKZ	U51477	Diacylglycerol kinase zeta	Intracellular signaling	48.1 ± 6.1	Y
EMILIN2	AF270513	Elastin microfibril interfacer 2	Cell adhesion	14.7 ± 3.7	Y
FOXC2	Y08223	Forkhead box C2	Transcription factor	5.9 ± 1.5	Y
GABBR1	Y11044	GABA receptor 1	Signal transduction	6.1 ± 2.0	Y
GABBR2	AF069755	GABA receptor 2	Signal transduction	2.8 ± 0.4	Y
GPR17	NM_005291	G-protein coupled receptor 17	Signal transduction	83.2 ± 12.5	Y
GZMA	NM_006144	Granzyme A	Apoptosis	2.1 ± 0.6	Y
IFNA1	NM_024013	Interferon alpha 1	Intracellular signaling	2.6 ± 1.0	Y
IL10RA	U00672	Interleukin 10 receptor alpha	Inhibition of proinflammatory cytokine synthesis	2.3 ± 0.2	Y
ITGB1	BC020057	Fibronectin receptor	Bacterial uptake	4.6 ± 0.7	Y
LCP2	NM_005565	Lymphocyte cytosolic protein 2	Immune response	37.5 ± 9.2	Y
MCAM	X68264	Melanoma cell adhesion molecule	Cell adhesion	4.7 ± 0.2	Y
MS4A1	M27394	Membrane-spanning 4-dmains	Immune response	9.6 ± 0.9	Y
PBX2	NM_002586	Pre-B-cell leukemia transcription factor 2	Transcriptional activator	3.0 ± 0.3	Y
PLTP	NM_006227	Phospholipid transfer protein	Lipid metabolism	3.6 ± 0.5	Y
RAB7	X93499	RAS related GTP binding protein	Vesicular transport regulation	3.4 ± 0.4	Y
RAB13	X75593	RAS related GTP binding protein	Small GTPase mediated signal transduction	7.5 ± 1.1	Y
RGS12	AF035152	Regulator of G-protein signaling 12	Negative regulation of G-protein signaling	3.0 ± 0.3	Y
RGS13	AF030107	Regulator of G-protein signaling 13	Negative regulation of G-protein signaling	2.6 ± 0.4	Y
S100A11	NM_005620	Calgizzarin	Motility, invasion & tubulin polymerization	9.6 ± 0.8	Y
TNFRSF17	Z29574	TNF receptor	Immune response	2.6 ± 0.3	Y
TUBB	AB062393	Tubulin beta	Microtubule based movement	4.0 ± 0.3	Y
YWHAZ	NM_003406	Tyrosine 3-monooxygenase	Signal transduction	4.3 ± 0.5	Y

Complemented 2D6 mutant had similar results to the wild-type bacterium. Y = Yes; N = No

### Macrophage gene expression analysis by quantitative real-time PCR

**Figure 1:**
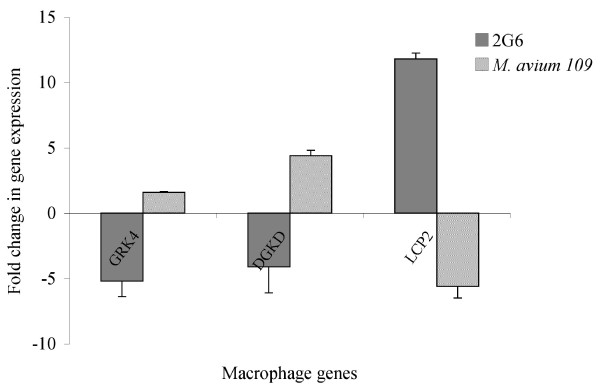
**Upregulation of U937 macrophage genes upon infection with *M. avium *or 2D6 mutant at 4 h, as determined by real-time PCR**. U937 were infected with MAC 109 or MAC 2D6. Four hours later, macrophage RNA was purified and used for real-time PCR to measure the expression of GRK4, DGKD and LCP2. The data represent the average of three independent experiments ± SD. A p < 0.05 for the three genes compared with the regulation in the other strain.

To confirm the changes in macrophage gene expression level upon infection with *M. avium *or its isogenic 2D6 mutant from the DNA microarray data findings, real-time PCR analysis was used to amplify GRK4 (G-protein coupled receptor kinase 4), DGKD (Diacylglycerol kinase delta), both upregulated in the wild-type but down-regulated in the 2D6 mutant infected macrophages, and LCP2 (Lymphocyte cytosolic protein 2) down-regulated in wild-type but upregulated in the 2D6 mutant. The gene β-actin was used as a positive control, while the uninfected cells were used as a negative control. As shown in Fig. 1, the two genes GRK4 and DGKD showed significant expression upon *M. avium *infection of macrophages, in contrast to infection by the 2D6 mutant. In addition, the LCP2 gene showed significant increased expression in macrophages upon infection with 2D6 mutant, in contrast to wild-type infected macrophages. None of the three genes showed upregulation in the uninfected negative control cells.

### Mass spectrometry analysis of isolated wild-type M. avium and 2D6 phagosomes

**Figure 2:**
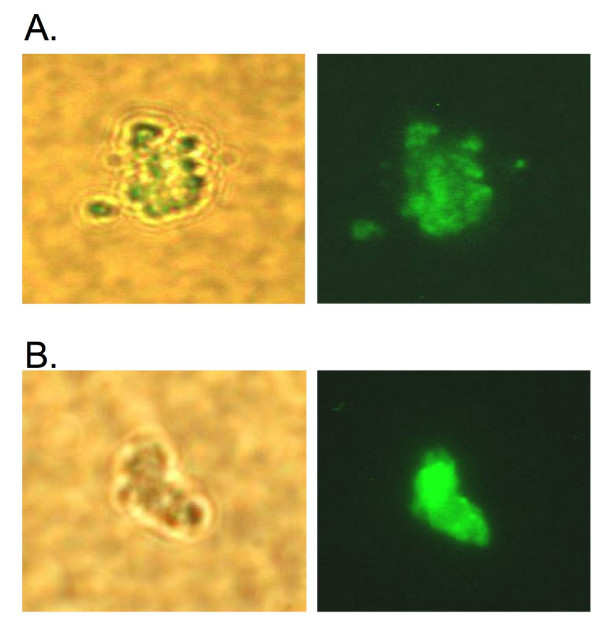
**Fluorescent microscopy images of isolated phagosomes showing phase contrast and fluorescein labeled images of: (A) *M. avium *and (B) 2D6 mutant**. *M. avium *vacuoles were purified according to method described in Materials and Methods. After centrifugation, purified phagosomes were analyzed under microscopy for purity.

Several of the genes differentially regulated in macrophages upon uptake of the wild-type bacterium or the 2D6 mutants are involved in signal pathways and G-protein receptor, which suggests an early diversification when comparing both bacterial strains. It was then hypothesized that MAV_2928 may be linked to the composition of the vacuole membrane. To examine the hypothesis, we first performed proteomic analysis in purified macrophage vacuoles. As shown in Fig. 2A and 2B, purified phagosomes of cells infected with MAC 109 or 2D6 were obtained. The MS/MS results of the phagosome membranes revealed several proteins with function in bacterial uptake, antigen presentation and recognition, Rab-interacting proteins, cytoskeleton and motor proteins, proteins involved in biosynthetic pathways, transcriptional regulation, and signal transduction proteins. Several of the proteins also have function as ion channels. A number of hypothetical proteins were also identified (Table 3). Some proteins observed were unique to MAC 109 or the 2D6 vacuoles at different time points. Together, these results suggest that the phagosomes with wild-type bacterium express a number of unique proteins, different from the vacuole of the 2D6 mutant. A proteomic analysis was attempted from vacuoles of uninfected macrophages. The results obtained were variable, probably reflecting the difference of the nature of the vacuoles (data not shown).

**Table 3:** List of phagosomal proteins identified by MS/MS post-infection with MAC 109 or 2D6 at different time points

		MAC 109			2D6 mutant		
Protein	Accession	(h)			(h)		
			
Bacterial Uptake	number	0.5	4	24	0.5	4	24
Complement c1q tumor necrosis factor related protein 5	Q9BXJ0	x	x			x	
Complement receptor 2	P20023			x			
Macrophage receptor MARCO	Q9UEW3	x	x			x	
Pulmonary surfactant protein D	P35247		x				
Scavenger receptor with C type Lectin type 1	Q9BYH7	x					
TNFRSF1A-associated via death domain/TRADD	Q15628				x		
Tumor necrosis factor receptor member 1A/TNFRSF1A	1NCFA						x
							
**Antigen Presentation & Recognition**							
Integrin alpha 1	P56199				x		
Integrin alpha IIb	P08514						x
MHC class I	Q95ID4						x
MHC class II	I54427						x
							
**Rab Interacting**							
EEA1	Q15075						x
Peroxin 5-related protein	Q81YB4	x			x		
Rabaptin 5	Q15276						x
							
**Cytoskeletal Proteins & Motors**							
Alpha actin	P62736				x		
Ankyrin 1	Q6S8J3				x		
Beta actin	Q96B34	x			x		
E-MAP-115	Q14244					x	
Keratin, type II cytoskeletal I	P04264	x			x		
Kinesin/KIF3	O14782						x
Kinesin family member 26A	Q8TAZ7	x					
L-plastin	P13796			x	x		
Myosin heavy chain non-muscle type A	P35579				x		
Myosin X with ATPase activity	Q9HD67				x	x	
RHOC	P08134				x		
TCP-1 zeta	P40227				x		
Tubulin alpha	Q71U36	x			x		
Tubulin alpha 2	Q13748	x					
Tubulin alpha 3	Q71U36	x					
Tubulin beta 2	P07437	x			x		
							
**Proteins in Biosynthetic Pathways**							
ABC transporter 2	Q9BZC7	x					
ADP-ATP translocase	P05141				x		
Aldoketo-reductase 3	Q9UFE1					x	
ALG12	Q9BV10		x				
ATP synthase	P06576		x	x			
Fatty acid synthase	P49327		x	x			
Fatty acid transporter member 1	Q6PCB7	x					
GAPDH	P04406			x			
Phosphatidyl inositol glycan class T	Q92643			x			
IMP dehydrogenase	P12268				x		
K-Cl co-transporter	T17231						x
Mitochondrial dicarboxylate carrier	Q9UBX3				x		
Neutral amino acid transporter	Q15758				x		
NUAK family, SNF1-like kinase 1	O60285					x	
PI4KII	Q9BTU6		x				
Pyruvate kinase	PS00110	x			x		x
Ribophorin II	Q5JYR7			x			
Trehalose precursor	O43280				x		
							
**Transcriptional Regulators**							
Lysine-specific histone demethylase 1	O60341		x				
CRSP complex subunit 2	O60244			x			
CREB binding protein	Q75MY6						x
DEAH box polypeptide 9	Q08211				x		
IFI 16	Q16666		x				
Msx2-interacting protein	Q96T58					x	
p-100 co-activator/SND1	Q7KZF4					x	
p-300/CBP associated factor	Q92831		x				
Zinc finger & BTB domain containing protein 4	Q9P1Z0		x				
Zinc finger protein 588	Q9UII5		x				
Zinc finger protein 43	P17038	x					
60 S acidic ribosomal protein P2	P05387				x		
60 S ribosomal protein L6	Q02878	x			x		
60 S ribosomal protein L9	P32969				x		
60 S ribosomal protein L14	P50914	x					
							
**Proteins Interacting with Signal Proteins**							
cAMP-specific 3',5'-cyclic phospho-diesterase	Q08493	x					
Calcium & lipid binding protein/NLF2	388125	x					
Chondroitin sulfate synthase 3	Q86Y52						x
CXC3C membrane-associated chemokine	P78423				x		
Doublecortin & CaM kinase like-3	Q9C098				x		
Dystrobrevin alpha	Q9Y4J8						x
Golgin subfamily A member 5	Q8TBA6						x
Microtubule associated-Ser/The kinase 3	O60307						x
Protein kinase A anchoring protein 9	Q99996		x				
Protein kinase N	Q8NF44		x				
Serine/theonine kinase 16	Q5U0F8			x			
TER ATPase	P55072				x		
							
**Ion Channels**							
Amiloride-sensitive cation channel	Q96FT7					x	
Voltage dependent-N-type calcium channel alpha 1B subunit	Q00975						x
Voltage dependent-T-type calcium channel alpha 1I subunit	Q9P0X4						x
							
**Other Proteins**							
AFG3 like protein 2	Q9Y4W6						x
APBB1	O00213				x		
Astrotactin 2	O75129		x				
Apoptotic chromatin-condensation inducer in the nucleus	Q9UKV3					x	
Clathrin heavy chain 1	Q00610				x		
Ephrin B3	Q15768	x					
48 kda histamine receptor subunit peptide 4	AAB34251			x			
HSP 60	P10809				x		
HSP 90	P07900				x		
Importin alpha 2	P52292				x		
Interferon regulatory factor 6	O14896						x
LRCH4/Ligand binding receptor	O75427						x
NADPH oxidase activator 1	Q2TAM1						x
Protein disulfide-isomerase A6	P07237	x			x		
p-53-associated parkin-like cytoplasmic protein/PARC	Q8IWT3					x	
Serine protease inhibitor	Q9NQ38		x				
Vitrin	Q6UX17						x
14-3-3 protein/Tyrosine 3-monoxygenase	P62258				x		
							
**Proteins with Unknown Function**							
Hypothetical protein DKFZp434A128	T34567						x
Hypothetical protein LOC136288	Q8NEG2						x
Hypothetical protein KIA1783	Q96JP2						x
Hypothetical protein KIAA1783	Q96JP2						x
Hypothetical protein FLJ32795	Q96M63					x	
Hypothetical protein FLJ42875	Q8N6L5					x	
Hypothetical protein FLJ45491	Q6ZSI8			x			
Hypothetical protein DKFZp434G131	Q9H0H4		x				
Hypothetical protein FLJ46534	Q6ZR97	x					
Hypothetical protein FLJ00361	Q8NF40	x					

Complemented 2D6 mutant had similar results to the wild-type bacterium. Y = Yes; N = No

### Immunofluorescence analysis

Many proteins identified in the mass spectrometry data have not previously been described in *M. avium *phagosomes. To confirm the data obtained by proteomic analysis, we used fluorescence microscopy to establish the presence of some selected proteins. One of the proteins, pulmonary surfactant protein D (SP-D), was observed to be selectively expressed in the MAC 109 phagosomes at 4 h time point but not in the 2D6 phagosomes. The second protein, T-type Ca^++ ^channel 1 alpha, showed selective expression in 2D6 phagosomes at 24 h after infection and not in the MAC 109 phagosomes.

**Figure 3:**
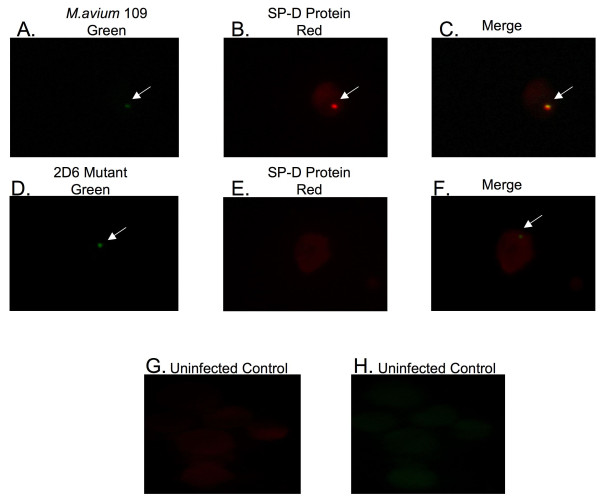
**Fluorescent microscopy images of U937 macrophages infected with *M. avium *MAC 109 strain or 2D6 mutant for SP-D protein expression**. (A-H) Phagocytic cells infected with fluorescein-labeled *M. avium *or 2D6 mutant were fixed and permeabilized at 4 h after infection. Antibody against SP-D protein was used and a second antibody labeled with Texas red was used. The arrows point to the green bacteria and red protein.

**Figure 4:**
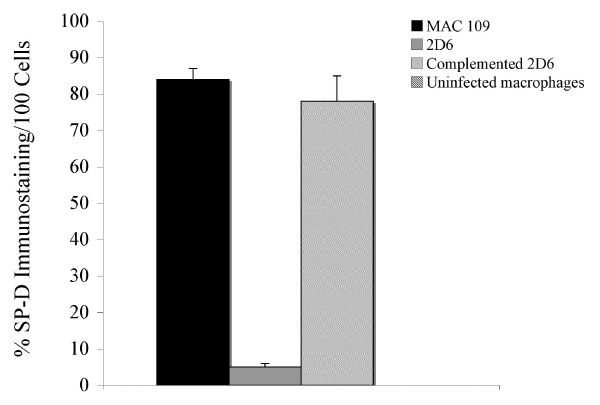
**Quantification of the SP = D protein expression assay in 100 U937 cells**. The numbers represent the mean ± SD of three experiments.

SP-D is a carbohydrate binding glycoprotein and has been shown to be involved in ligand binding and immune cell recognition [18]. To confirm the expression of SP-D on MAC 109 vacuoles at 4 h time point, cells were infected in parallel with fluorescein-labeled bacteria (MAC 109 and 2D6) for 4 h. The SP-D protein was observed to be present in nearly all on the MAC 109 vacuoles (Fig. 3, A-C). In vacuoles with the 2D6-mutant, as well as in uninfected, macrophage, no SP-D was co-localized with *M. avium *(Fig. 3, D-F & Fig. 3, G-H, respectively). Quantification is shown in Fig. 4. This finding confirmed our data that SP-D is expressed in MAC 109 phagosomes but not in 2D6 mutant phagosomes at 4 h time point. The protein was also seen in the membrane of infected macrophages.

**Figure 5:**
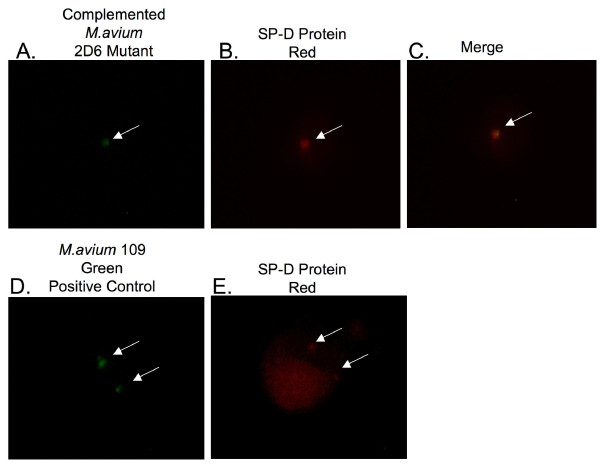
**Fluorescent microscopy images of U937 macrophages infected with fluorescein-labeled complemented *M. avium *2D6 mutant**. The SP-D protein is shown in red. Arrows point to bacteria (green) and SP-D protein (red). SP-D is present in macrophages infected with the MAC 104 strain and absent in the 2D6 mutant-infected macrophages.

To investigate whether the complemented *M. avium *2D6 mutant phagosomes showed similar protein expression as that of wild-type, we infected the cells with 2D6 complemented bacteria [11] for 4 h, with MAC 109 as a positive control. The vacuoles containing the complemented *M. avium *2D6 mutant showed expression of SP-D protein (Fig. 5A-5C) similarly to vacuoles containing the wild-type bacterium (Fig. 5D and 5E), though the percentage of infected cells showing the protein expression was 15% less than in macrophages infected with the wild-type bacterium. Quantification of expression is shown in Fig. 4.

**Figure 6:**
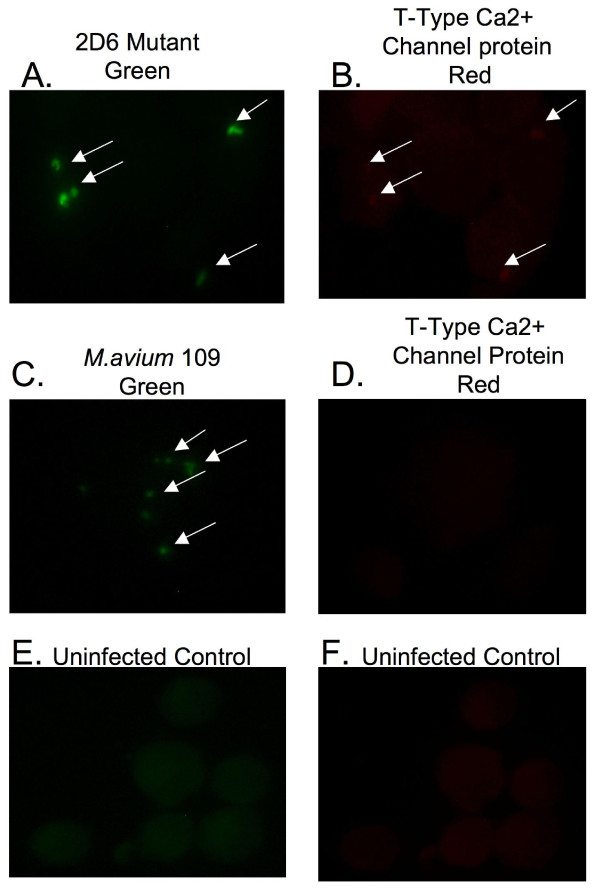
**Fluorescent microscopy images of U937 macrophages infected with fluorescein-labeled *M. avium *MAC 109 strain or 2D6 mutant**. Macrophages were fixed and permeabilized 24 h after infection. Antibody anti-T-type Ca^++ ^channel protein was used for 1 h, washed, and second antibody labeled with Texas red was applied for an additional hour. The arrows point to the green bacteria and red protein (A-F).

T-type Ca^++ ^channel is an integral membrane protein, which controls the rapid entry of Ca^++ ^into excitable cells, and is activated by CaM-Kinase II (Swiss-Prot database). To verify our initial observation by MS/MS, we carried out parallel infection assays with fluorescein-labeled 2D6 and MAC 109 bacteria for 24 h. As shown in Fig. 6A and 6B, the majority of the cells infected with 2D6 mutant showed T-type Ca^++ ^channel protein staining; whereas, those infected with the wild-type MAC 109 and uninfected control U937 cells failed to express the protein (Fig. 6C and 6D, Fig. 6E and 6F, respectively). The observation was in agreement with the proteomic data showing that T-type Ca^++ ^channel is expressed in mononuclear phagocytes infected with 2D6 attenuated mutant, but not when infected with MAC 109.

**Figure 7:**
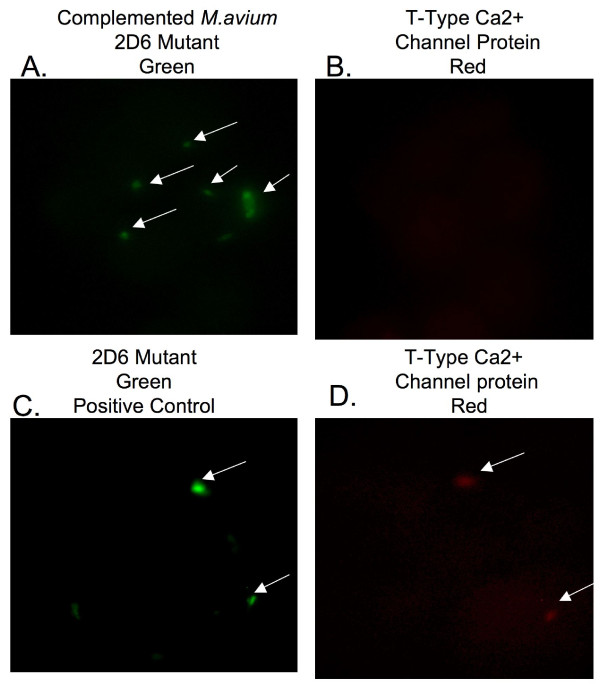
**Fluorescent microscopy images of U937 macrophages infected with fluorescein-labeled complemented 2D6 mutant**. The T-type Ca^++ ^channel protein is labeled by antibody conjugated with Texas red. The arrows point to the bacteria (green) and T-type Ca^++ ^channel protein (red) (A-D).

**Figure 8:**
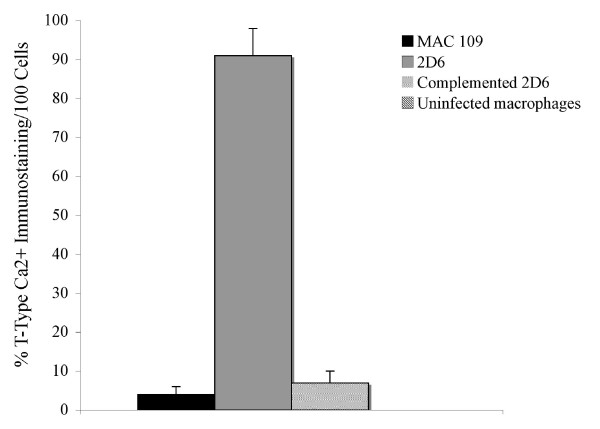
**Quantification of the T-type Ca^++ ^channel protein assay in 100 U937 cells**. The numbers represent the mean ± SD of the three experiments. * p < 0.05.

To determine whether the phagosomes of macrophages infected with the complemented *M. avium *2D6 mutant phagosomes failed to express the T-type Ca^++ ^channel, mononuclear cells infected with complemented *M. avium *2D6 bacteria and 2D6-attenuated mutant were evaluated. As shown in Fig. 7A and 7B, vacuoles with the complemented bacteria, in contrast to the 2D6 mutant (Fig. 7C and 7D), did not express T-type Ca^++ ^channel protein. Quantification of the T-type Ca^++ ^channel protein in macrophages infected with MAC 109, the 2D6 mutant and complemented 2D6 strain are shown in Fig. 8.

**Figure 9:**
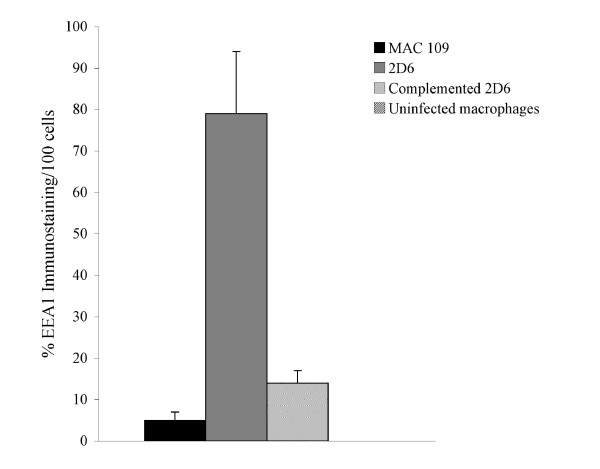
**Quantification of the expression of labeled antigen by fluorescence microscopy in 100 U937 cells**. EEA1 at 24 h (p < 0.05 for the comparison between MAC 109 and complemented 2D6 strain).

The expression of EEA-1, CREB-1, and TNFRI were also quantified by immunofluorescence microscopy, as shown in Fig. 9-Fig. 11. Expression of EEA-1, CREB-1 and TNFRI proteins was selectively observed after macrophage infection with 2D6 bacteria but not in the vacuoles of macrophages infected with the wild-type bacterium. Western blot analysis showed that EEA-1 and CREB-1 proteins were only expressed in vacuoles occupied by the 2D6 mutant and not the wild-type bacteria. MARCO, a protein shown by the mass spectrometry to be expressed differently in macrophages infected by the mutant and wild-type bacterium, was present in the vacuole membrane of the wild-type bacterium at 30 min but not in 2D6 mutant vacuole. The expression decreased significantly in the vacuole of the wild-type *M. avium *at 24 h but increased significantly in the vacuoles of 2D6 mutants (Fig. 12).

**Figure 10:**
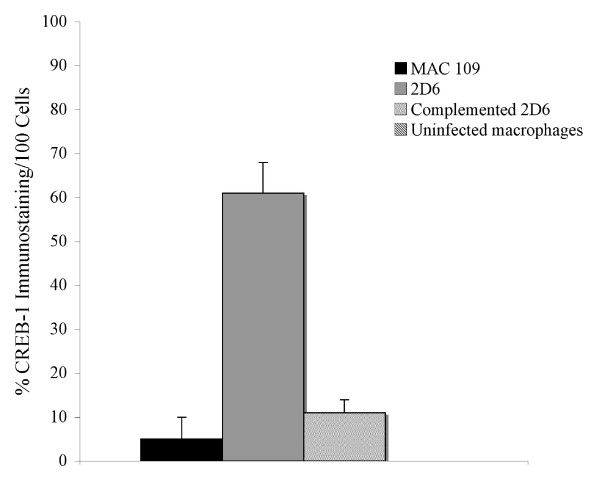
**Quantification of the expression of labeled antigen by fluorescence microscopy in 100 U937 cells**. CREB-1 at 24 h (p < 0.05 for the comparison between MAC 109 and complemented 2D6 strain).

**Figure 11:**
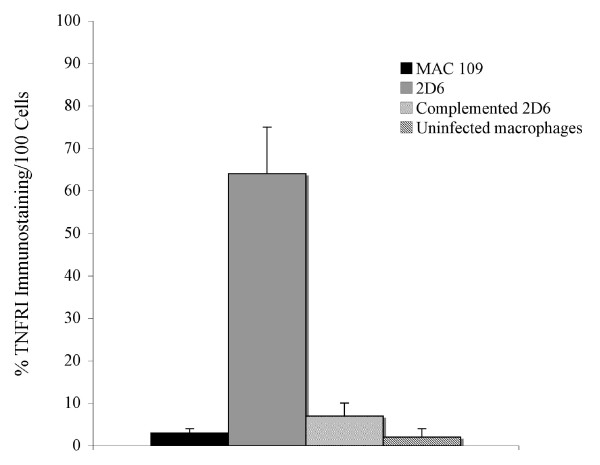
**Quantification of the expression of labeled antigen by fluorescence microscopy in 100 U937 cells**. TNFRI at 24 h (p < 0.05 for the comparison between MAC 109 and complemented 2D6 strains and 2D6 strain). The assays were repeated three times.

**Figure 12:**
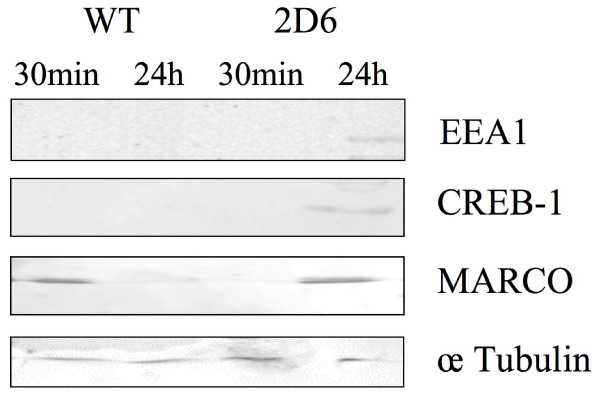
**Western blot of vacuole membrane using antibodies against EEA-1, CREB-1, MARCO and α-tubulin antigens**. The assay was repeated twice. Comparison of antigen expression between vacuole membrane of macrophages infected with wild-type bacterium MAC 109 and 2D6 mutant were carried out at 30 min and 24 h. Specific methods are described in the text.

### X-ray microscopy measures of intravacuolar concentrations of elements

Because the changes in the vacuole membrane might translate into changes in the vacuole environment, we carried out hard x-ray microscopy to evaluate the level of single elements within the bacterial vacuole. We observed that, at 1 h after infection, the concentration of Mn^++ ^and Zn^++ ^were significantly higher in vacuoles occupied by the 2D6 mutant than in vacuoles of the wild-type bacterium. At 24 h, the concentration of Mn^++ ^remained similar in vacuoles with the 2D6 mutant but decreased in vacuoles of the wild-type organism. The concentrations of Ca^++ ^and K^+ ^also decreased over time in 2D6 mutant vacuoles, becoming significantly different from the wild-type bacterium (Table 4). The concentration of Zn^++^, while still significantly different between the wild-type bacterium and the 2D6 mutant, also decreased over time (Table 4). The concentration of iron in the vacuole of 2D6 mutant did not differ from the concentration in vacuoles with the wild-type bacterium.

**Table 4:** Concentrations of single elements in phagosomes of macrophages infected with *M. avium *wild-type (WT) or 2D6 mutant

Element (Unit)	WT	2D6	WT	2D6
	
	1 hour		24 hours	
P (CPM)	0.013964	0.0144769	0.010927	0.0072144
	(p > 0.05)		(p > 0.05)	
S (CPM)	0.01848	0.0210543	0.035871	0.0099751
	(p > 0.05)		(p > 0.05)	
Cl (CPM)	0.151509	0.2305818	0.244938	0.1115413
	(p > 0.05)		(p > 0.05)	
K (μg/cm^2^)	0.143707	0.3204288	0.021604	0.1759281
	(p = 0.05)		(p = 0.0009)	
Ca (μg/cm^2^)	6.5 × 10^-5^	0.0329014	0.010014	0.0224007
	(p = 0.821)		(p = 0.00492)	
Mn (μg/cm^2^)	6.5 × 10^-5^	0.00018	0.000133	8.204 × 10^-5^
	(p = 0.0308)		(p = 0.302)	
Fe (μg/cm^2^)	0.00167	0.0054284	0.006516	0.0022057
	(p = 0.3025)		(p = 0.12196)	
Cu (μg/cm^2^)	0.000183	0.1394013	0.000112	0.0148152
	(p > 0.05)		(p > 0.05)	
Zn (μg/cm^2^)	0.00088	0.015652	0.000792	0.005898
	(p = 0.00517)		(p = 0.02767)	

Complemented 2D6 mutant had similar results to the wild-type bacterium. Y = Yes; N = No

## Discussion

*M. avium*, like *M. tuberculosis*, primarily infects the host mononuclear phagocytes. Targeting mononuclear phagocytes and being able to survive within the presence of efficient mechanisms of macrophage subversion, evolved by virulent.

In *M. tuberculosis*, PE-PGRS and PPE are two families of glycine-rich protein which constitute approximately 10% of the *M. tuberculosis *genome. Recent reports have suggested that these two gene families might be involved in antigen variation, eukaryotic cell binding, survival within macrophages and persistence in granulomas [19,20]. Richardson and colleagues (2001) showed that a PPE protein (Rv1917) is expressed on the bacterial surface. Using signature-tagged mutagenesis, Camacho and colleagues identified a PPE gene (Rv3018c) associated with *M. tuberculosis *virulence *in vivo *[21]. In addition, Ramakrishnan and colleagues observed that inactivation of PE-PGRS gene in *Mycobacterium marinum *resulted in attenuation of bacterial virulence in macrophages [19]. In a recent report, Li and colleagues [11] demonstrated that an *M. avium *strain lacking a functional PPE protein, MAV_2928 (homologue to Rv1787), is attenuated *in vivo *and fails to inhibit both acidification of the vacuole, as well as phagosome-lysosome fusion. *Mycobacterium avium *MAV_2928 transposon mutant had comparable ability to enter the mononuclear phagocytes as the wild-type bacterium. The expression of MAV_2928 was noted following uptake of the wild-type bacterium by macrophages but not in culture media, suggesting a possible participation in the early events of the intracellular stage and possibly in phagosome formation [11].

The gene MAV_2928 is part of an *M. avium *chromosomal region with five PPE and PE genes, adjacent to the region homologous to the RD5 region in *M. tuberculosis*. The organization of this region suggests the existence of three promoters, one upstream of MAV_2928 inactivated in the 2D6 mutant, one between the second, and the third genes and another between the fourth and fifth genes in the downstream region [11]. This specific region is also upstream of a region homologous to the RD1 region of *M. tuberculosis*. A PPE gene adjacent to the RD1 region in *M. tuberculosis *has been suggested to be associated with the transport of proteins [15]. Because MAV_2928 is co-transcribed with MAV_2929, it is possible that some of the findings are due to the downstream gene. Complementation of the 2D6 mutant, however, has shown that most of the function lost with the inactivation of MAV_2928 is recovered [11]. Interestingly, MAV_2925 has a high degree of homology with MAV_2928, but, based on the phenotype obtained with the inactivation of MAV_2928, we assume that the genes probably have unique functions.

Usually, upon bacterial uptake, a macrophage undergoes a series of events specifically designed to eliminate the engulfed microorganism. These include induction of reactive oxygen and nitrogen intermediates, gradual acidification of the phagosome, phagosome-lysosome fusion which loads the resulting compartment with acidic proteolytic enzymes, and antigen processing and presentation. The resulting lethal environment effectively kills the majority of the ingested bacteria. Pathogenic mycobacterial phagosomes, in contrast, show incomplete luminal acidification and absence of mature lysosomal hydrolases [22]. Malik *et al*. [10,23,24] suggested that *M. tuberculosis *manipulation of calcium is in part responsible for the phagosome maturation arrest. The pathogenic mycobacterial phagosome has been shown to alter the trafficking of the plasma membrane markers, including MHC molecules [25], EEA-1 and LAMP-1 [6]. *M. tuberculosis*-related blocking of phagosome maturation in macrophages appears to take place between the maturation stages controlled by early endocytic marker Rab5 and late endocytic marker Rab7 [6]. The published data indicate that virulent mycobacterial phagosomes are selective in their fusion with various cytoplasmic organelles and do not mature into a phagosome-lysosome. Currently unknown is whether this ability to impact the docking and incorporation of proteins in the phagosome membrane is due completely, or partially, to the proteins that form the phagosome membrane is currently unknown. It is a plausible possibility. This interpretation could explain the differences between the vacuole proteomic between both bacterial strains.

Based on the results obtained in the macrophage transcriptome following infecting with *M. avium *or the 2D6 clone, it is clear that the mutant stimulates membrane-based signals and receptors that are bypassed by the wild-type bacterium.

Mass spectrometry analysis of the phagosomal proteins of 2D6 mutant and the wild-type bacterium yielded several differences in the protein expression in the vacuole membrane. For example, expression of EEA-1 and Rab5 effectors was seen on 2D6 phagosomes but not on the wild-type phagosomes, which is in agreement with the observation reported by Fratti *et al*. and Via *et al*. [6,26]. The upregulation of Rab7 on the 2D6-infected macrophages indicates that the 2D6 mutant expresses late endosome markers and undergoes phagolysosome fusion [11].

A relatively large body of published data suggests the role of complement receptors CR1, CR3 and CR4 [27] and a mannose receptor [27] in the uptake of *M. tuberculosis *by macrophages. It has been shown that CR3 is one of the main receptors involved in phagocytosis of *M. avium *by macrophages and monocytes [28,29]. The CR2 was identified among various receptors on *M. avium *phagosomes. Studies have suggested an important role of CR1/2, CR3 and CR4 in host defense against *Streptococcus pneumoniae *infections [30]. Functional studies have demonstrated that CR2 mediates tyrosine phosphorylation of 95 kDa nucleolin and its interaction with phosphatidylinositol 3 kinase [31].

Surfactant-associated proteins A and D (SP-D) are pulmonary collectins that bind to bacterial, fungal and viral pathogens and have multiple classes of receptors on both pneumocytes and macrophages [32]. In addition, they act as chemoattractant to phagocytes. Surfactant proteins A and D (SP-A and -D) participate in the innate response to inhaled microorganisms and organic antigens and contribute to immune and inflammatory regulation within the lung [33]. Ferguson and colleagues showed that SP-D binds to *M. tuberculosis*, resulting in decreased uptake and inhibition of bacterial growth [34]. The presence of SP-D in phagosomes MAC 109 suggests a host attempt to eliminate the pathogen. Surfactant protein A (SP-A) expressed on *M. tuberculosis *vacuoles has been shown to be involved in enhancing the uptake of bacteria by macrophages [35-37].

The lack of MHC class II molecule expression in *M. avium *phagosomes, and its presence in the attenuated 2D6 mutant phagosomes in our data, is in agreement with the above findings that MHC class II molecules are down-regulated upon mycobacterial infection [38-40]. The MHC class I molecules are involved in presentation of the antigens located in the cytoplasm. The fact that MHC class I molecules were found on 2D6 mutant phagosomes, at 24 h time point, may reflect altered trafficking by the bacteria. In addition, MHC class I expression at early time points on the phagosome would suggest that the protein being present on the cell surface, during phagocytosis, would have been ingested upon during vacuole formation. The presence of MHC class I molecules on the 2D6 phagosomes could also be due to the fact that mycobacterial antigens are processed by MHC class I [41]. The MHC class I has been reported to be present on latex bead phagosomes [42].

Several proteins not previously shown to be associated with the mycobacterial phagosomes were identified in the phagosomal preparations. Because we could not completely rule out the possibility of contamination of the phagosome preparations with other organelles, which indeed is a limiting factor of most subcellular fractionation techniques, we confirmed the findings by identifying proteins by fluorescence microscopy and Western blot. Recent studies on *Legionella *and *Brucella *have shown that these organisms reside in compartments displaying features of endoplasmic reticulum (ER) [43]. In addition, there is evidence of recruitment of endoplasmic reticulum (ER) to nascent phagosomes containing inert particles or *Leishmania *and having a major contribution to the phagosomal membrane [16]. This explains how antigens of vacuolar pathogens are presented to T lymphocytes via MHC class I machinery located on ER. Considering this information, it would be plausible to find ER particles on mycobacterial phagosomes. Some of the mitochondrial proteins, such as ATP synthase and HSP60 found in our preparations, have also been shown to be present in latex bead containing phagosomes [42].

A recent report on the elemental analysis of *M. avium *phagosomes in Balb/c mouse macrophages revealed high concentrations of potassium and chlorine at 24 h time point and correlated it to the microbiocidal killing similar to that observed in neutrophils [44]. The increase in expression of CHP (potassium channel regulator) in the 2D6-infected macrophages, added to the finding that K-Cl co-transporter is also increased (proteomic results) on the 2D6 mutant phagosomes at 24 h time point, could support, at least in part, the above published report, since the 2D6 mutant is unable to survive within the macrophages [11]. Therefore, there is a possibility that K-Cl transporter and CHP could be involved in the augmentation of the potassium and chlorine concentrations in the phagosome, leading to mutant killing, but this will have to be tested in future work. Because of the observed difference in vacuole membrane between the two tested bacterial strains, it was hypothesized that the difference might impact the content of the metals in the vacuole environment.

Measurement of the intravacuolar concentration of single elements demonstrates that the 2D6 mutant's vacuole is depleted of several important elements at 24 h after infection. The decrease in the intravacuolar concentrations of Ca^++ ^and Zn^++ ^suggests that the wild-type bacteria are capable of retaining the elements, but the PPE mutant is not, probably indicating that the mutant cannot suppress the transport mechanisms or cannot continue to induce uptake of the metals.

We studied protein expression of the mycobacterial phagosome and compared it to a isogenic mutant. We identified several proteins, either previously described or not reported to be present on the phagosomes. The modifications appear to have a significant effect on the intravacuolar environments. Nonetheless, the use of the MAV_2928 mutant established the possibility that one protein may have key function in modulating the formation of the phagosome, perhaps by altering initial events. Alternatively, the PPE-PE operon may be part of a complex system influencing or impacting the expression of other bacterial genes or involved in the transport of bacterial proteins. Change in single element concentrations in the bacterial environment can have significant effect on gene regulation [45]. Future studies will address some of the differences found and will possibly provide insights into the mechanisms of pathogenesis and survival of mycobacteria inside the host.

## Conclusion

1. Inactivation of MAV_2928 alters early stages of macrophage transcription in response to MAC infection.

2. Absence of MAV_2928 affects the concentration of materials inside the MAC vacuole, indicating changes in the transport mechanisms.

3. Investigation of the phagosome membrane components revealed unexpected results for the action of only one protein, suggesting that MAV_2928 may be involved in the transport of other proteins into the host cell.

4. Future studies will attempt to identify proteins that are secreted by the PPE MAV_2928-dependent mechanism.

## Methods

### Bacterial strains and growth conditions

*Mycobacterium avium *strain 109 (MAC 109), a virulent strain in mice initially isolated from blood of a patient with AIDS, was cultured from 20% glycerol stock onto Middlebrook 7H11 agar supplemented with oleic acid, albumin, dextrose and catalase (OADC; Hardy Diagnostics, Santa Maria, CA) at 37°C for 21 days. For the assays, bacteria were suspended in Hank's buffered salt solution (HBSS) and passed through a 26-gauge needle 10 times to disperse clumps. The suspension was then allowed to rest for 5 min and the upper half was used for the assays. The bacterial concentration was adjusted to 1 × 10^8 ^bacteria ml^-1 ^using a McFarland standard. Microscopic observations of the suspensions were carried out to verify dispersion of bacteria. Only well dispersed inocula were used in the described experiments. The 2D6 mutant was cultured from 20% glycerol stock on Middlebrook 7H11 agar containing 400 μg/ml kanamycin. The 2D6 mutant suspension was made as described above. The complemented 2D6 strain [11] was also cultured from 20% glycerol stock and grown on Middlebrook 7H11 agar plates containing 200 μg/ml apramycin [11].

### Cells and culture conditions

Human monocytic cell line U937 (ATCC CRL-1593.2) was cultured in RPMI-1640 (Gibco Laboratories) supplemented with 10% heat-inactivated fetal bovine serum (FBS; Sigma Chemical), 2 mM L-glutamine. The U937 cells were used between passages 15 to 20 and concentrations of 7 × 10^6 ^were seeded in 75 cm^2 ^flasks. The cell line was chosen because of convenience, since the strains grow similarly in U937, THP-1 and monocyte-derived macrophages. The U937 was the cell line that allowed the purification of the greater number of vacuoles [11]. The cells were grown to 90-100% confluency and allowed to differentiate overnight by incubation with 500 ng ml^-1 ^phorbol 12-myristate 13-acetate (PMA; Sigma). Human monocyte-derived macrophages and U937 were shown to behave similarly when infected with *M. avium *wild-type and 2D6 mutant [11]. The MAC 109 or 2D6 mutant were added to the monolayers at a multiplicity of infection (MOI) of 10, and the infection was allowed to take place for 2 h at 37°C in 5% CO_2_. The supernatant was then removed and the cell monolayer was washed three times with HBSS. The tissue culture medium was then replenished.

### RNA extraction

For the DNA microarray, the U937 infection assay for MAC 109, 2D6 mutant, and the complemented 2D6 mutant followed by RNA isolation was carried out as described previously [46]. Briefly, U937 monolayers of approximately 10^8 ^cells were infected with MAC 109 or 2D6 (1 × 10^8 ^concentration) for 4 h. The cells were washed to remove extracellular bacteria and total RNA was isolated using Atlas Pure Total RNA Labeling System (Clontech Laboratories, Palo Alto, CA) according to the manufacturer's instructions. The resultant RNA was treated with DNase for 30 min at 37°C followed by phenol-chloroform extraction and precipitation with ethanol. The RNA was run on 1% denaturing agarose gel and quantified by UV spectrometer at 260/280 nm. RNA was then submitted to analysis using the bioanalyzer at the Center for Genome and Biotechnology Research at OSU.

To confirm the expression, as well as to determine the relative transcriptional levels of G-protein coupled receptor kinase 4 (GRK-4), diacylglycerol kinase delta (DGKD) and lymphocyte cytosolic protein 2 (LCP2) by real-time PCR, similar U937 infection assay was performed as described above and modifications in the RNA extraction method were made. After 4 h, the monolayers were washed with HBSS, scraped and collected in a 50 ml falcon tube and placed on ice. The cells were centrifuged at 500 rpm for 5 min to remove any residual extracellular bacteria. Then, 2 ml of Trizol (Invitrogen, Carlsbad, CA) was added to the falcon tube. The suspension was then passed 20 times through a 21-gauge needle to lyse the mononuclear cells. The lysate was then centrifuged at max (14,000) rpm at 4°C. The supernatant was then transferred to heavy Lock Gel I (Eppendorf, NY), and to it chloroform:isoamyl alcohol (24:1) (Sigma) was added and mixed. After centrifugation, the aqueous phase was precipitated in isopropanol followed by 75% ethanol wash to remove isopropanol. The DNase treatment of total RNA was carried out before probe synthesis using the protocol described by the Atlas Pure Total RNA Labeling System (Clontech, Mountain View, CA). The quality of RNA was verified on a 1% denaturing agarose gel, and the concentration was calculated based on the absorbance at 260 nm.

The cDNA was synthesized as per the protocol described by Invitrogen (Carlsbad, CA). Total RNA (5 μg) with oligo(dT)_20 _and dNTP mix was incubated at 65°C for 5 min and cooled on ice for 1 min. For each total RNA sample, 10 μl cDNA synthesis mix was made: 10× RT buffer, 25 mM MgCl_2_, 0.1 M DTT, 40 U/μl RNaseOUT and 200 U/μl Superscript III RT. The samples were mixed gently and collected by brief centrifugation. Then, the samples were incubated in a thermal cycler at 42°C for 50 min and the reaction was terminated at 70°C for 15 min and cooled on ice. Finally, the reactions were collected by brief centrifugation, and 1 μl of RNase H was added to each sample and incubated for 20 min at 37°C. The cDNA prepared was used for real-time PCR.

### DNA microarray

The ^32^P-labeled cDNA probes were prepared using the Atlas Pure Total RNA Labeling System (Clontech Laboratories) as previously described [46]. This array was the only one available commercially when the experiments were performed. In brief, 5 μg of total RNA was reverse transcribed using the primer mix supplied with each array. The mixture was heated to 65°C for 2 min in a PCR thermal cycler, followed by 50°C for 2 min in presence of a master mix containing 5× Reaction buffer, dNTP, and dATP. The DTT and MMLV reverse transcriptase was added, mixed and incubated for 25 min at 50°C. Then, 10× termination mix was added to end the reaction. Unincorporated nucleotides were removed using a Nucleospin Extraction Spin Column (Clontech Laboratories, Palo Alto, CA) as per the manufacturer's instructions. Scintillation counting was done to measure the incorporation of radionucleotide into the probe.

The Clontech Human Nylon Filter Arrays (Clontech Laboratories), containing DNA sequences for 1,500 genes, were prehybridized in 5 ml of Express-Hyb solution supplemented with 0.5 mg salmon testes DNA at 68°C for 30 min. The radiolabeled cDNA probe was heated in a boiling water bath for 2 min, followed by 2 min on ice. Then it was added to the hybridization solution and allowed to hybridize to the filter array overnight. The membranes were washed in SSC plus 0.1% sodium dodecyl sulphate (SDS) at 68°C for 30 min and further rinsed in SSC for 5 min at room temperature. Next, the filters were wrapped in plastic wrap and exposed to a phosphor imaging screen for 24 h. Analysis of the phosphor imaging screens was done by using a phosphor imager (Perkin Elmer, Boston, MA) and AtlasImage 2.0 software. Global normalization method was used, by the background subtraction method followed by SAM analysis. For most of the genes, a Q value (percent change that the gene is false-positive) of 5% was used as the cut-off value. The quality of the hybridization signals was assessed using scatter plot analysis of replicate samples, as well as by calculating the coefficient of variance. Only samples with hybridizations with high correlation levels (p > 0.9) among replicates were used for subsequent analysis. The following genes were used as housekeeping genes: glyceraldehyde 3-phosphate dehydrogenase (GAPDH), tubulin alpha 1 (TUBA1), hypoxanthine-guanine phosphoribosyltransferase 1 (HPRT1), major histocompatibility class 1 C (HLAC), beta-actin (ACTB) and 23-kDa highly basic protein (PRL13A). Only genes that showed differential expression at least by two-fold were incorporated in the results.

### Real-time PCR

Genes were chosen randomly for real-time PCR analysis, and SYBR technology was used. Run protocol for the LightCycler was as follows: denaturation 95°C for 5 min; amplification and quantification repeated for 35 times: 95°C for 30 sec, 59°C for 30 sec and 72°C for 1 min with one fluorescence measurement followed by 72°C for 5 min and 4°C. Table 5 shows the sense and anti-sense plasmid.

**Table 5:** Sense and antisense primers for real-time PCR

Target	Primers	PCR product (bp)
β-actin	5'-TGATGGTGGGCATGGGTCAGA-3' 5'-CCCATGCCAATCTCATCTTGT-3'	800
GRK4	5'-AATGTATGCCTGCAAAAAGC-3' 5'-GATTGCCCAGGTTGTAAATG-3'	235
DGKD	5'-CTCGGCTTACGGTTATTCCAG-3' 5'-CCATCTCCATCTTCAGCCTCC-3'	656
LCP2	5'-CACTGAGGAATGTGCCCTTTC-3' 5'-GTGCCTCTTCCTCCTCATTGG-3'	408

Complemented 2D6 mutant had similar results to the wild-type bacterium. Y = Yes; N = No

The threshold cycle (Ct) is defined as the fractional cycle number at which the fluorescence reaches 10× the standard deviation of the baseline and was quantified as described in User Bulletin #2 for ABI PRIMS 7700 sequence detection system (ABI). The fold change in gene expression was determined using an amplification-based strategy. For each gene amplification, before calculating the fold change, the Ct values were normalized to the Ct of β-actin using the following formula:

Quantitative analysis was performed using iCycler I software (BIORAD, Hercules, CA). A relative quantification was used in which the expression levels of macrophage target genes were compared to data from a standard curve generated by amplifying several dilutions of a known quantity of amplicons. Real-time PCR efficiency was determined using a dilution series of cDNA template with a fixed concentration of the primers. Slopes calculated by the LightCycler software were used to calculate efficiency using the following formula: E = 10^(-1/slope)^. These calculations indicated high real-time efficiency with a high linearity. Because expression of β-actin is constant, independent of conditions, target genes from both control and experimental groups were normalized to the expression level of the β-actin gene.

### Phagosome isolation and microscopy

Phagosomes containing *M. avium *109 and 2D6 mutant were isolated according to a protocol described previously [4], with minor modifications [11]. Briefly, infected macrophages were added to homogenization buffer and scraped from tissue culture flasks. The cells were lysed by approximately 30 passages through a tuberculin syringe (at least 90% of the cells were lysed), and the lysate was carefully deposited over a 12% to 15% sucrose gradient. The preparation was then centrifuged at 2000 rpm for 40 min at 4°C. After centrifugation, the interface was collected and centrifuged in 10% Ficoll solution at 2500 rpm for 40 min at 4°C. A small pellet containing phagosomes was visible at the bottom of the tube. Phagosomes were analyzed for purity visually on glass slides by staining MAC 109 or 2D6 prior to infection with 10 μg/ml N-hydroxysuccinimidyl ester 5-(and-6)-carboxyfluorescein (NHS-CF; Molecular Probes, Eugene, OR) for 1 h at 37°C. Phagosomes containing live *M. avium *or 2D6 showed green fluorescein stain when observed under 100× oil immersion (Leica DMLB Scope, Spot 3^rd ^Party Interface; Diagnostics Instruments Inc.). Approximately 98% of the phagosomes observed showed bacteria in them.

### Mass spectrometry

The phagosome samples were run by lc/ms-ms using a Waters (Milford, MA) NanoAcquity HPLC connected to a Waters Q-TOF Ultima Global. Phagosome preparation, isolated as described above, was treated using the In-Gel Tryptic Digestion Kit from Pierce (Rockford, IL), according to instructions provided by the manufacturer. Briefly, the phagosome preparation was treated with activated trypsin for 15 min at room temperature. The suspension was transferred to 37°C for 4 h. The digestion mixture was then placed in a clean tube. To further extract peptides, 10 μl of 1% trifluoroacetic acid was added for 5 min. Five microliters of a sample was loaded onto a Waters Symmetry C18 trap at 4 μl/min, then the peptides were eluted from the trap onto the 10 cm × 75 μm Waters Atlantis analytical column at 350 nl/min. The HPLC gradient went from 2% to 25% B in 30 min, then to 50% B in 35 min, then 80% B in 40 min and held there for 5 min. Solvent A was 0.1% formic acid in water, and B was 0.1% formic acid in acetonitrile. Peptide "parent ions" were monitored as they eluted from the analytical column with 0.5 sec survey scans from m/z 400-2000. Up to three parent ions per scan with sufficient intensity and 2, 3, or 4 positive charges were chosen for ms/ms. The ms/ms scans were 2.4 sec from m/z 50-2000.

The mass spectrometer was calibrated using the ms/ms spectrum from glu-fibrinopeptide. Masses were corrected over the time the calibration was used (one day or less), using the Waters MassLynx DXC system.

The raw data were processed with MassLynx 4.0 to produce pkl files, a set of smoothed and centroided parent ion masses with the associated fragment ion masses. The pkl files were searched with Mascot 2.0 (Matrix Science Ltd., London, UK) database searching software, using mass tolerances of 0.2 for the parent and fragment masses. The Swiss Prot database was used, limiting the searches to human proteins. Peaks Studio (Bioinformatics Solutions Inc., Ontario, Canada) was also used to search the data, using mass tolerances of 0.1, and the IPI human database.

The proteomic analysis was compared to the protein profile of bacteria grown on 7H10 plates. Then, if the protein expression was increased or decreased at least 1.5-fold, the data were included. Proteins or peptides to be included in the analysis had to be present in both runs. Proteins present in only one run were not included.

### Immunofluorescence analysis

Because some of the proteins identified in the phagosomes have not been previously described as part of the vacuole membrane, we attempted to confirm their presence by using immunofluorescence. Primary antibodies against pulmonary surfactant protein D (SP-D), T-type Ca^++ ^alpha1I protein, EEA-1, CREB-1, MARCO and α-tubulin were purchased from Santa Cruz Biotechnology, Santa Cruz, CA. Primary antibodies used were from rabbit, except the goat anti-T-type Ca^++ ^alpha1I. Secondary antibodies were Texas-Red conjugates (TR) and included donkey anti-rabbit IgG-TR (Amersham Biosciences, Piscataway, NJ) and mouse anti-goat IgG-TR (Santa Cruz Biotechnology, Santa Cruz, CA). The two-chamber slides from Nalge Nunc (Rochester, NY) were employed for macrophage monolayer preparation and fluorescence microscopy. The numbers of U937 cells were determined in a hemocytometer before seeding. A total of 5 × 10^5 ^cells were added in each tissue culture well of the two-chamber slides and were differentiated with 2 μg/ml of PMA overnight. The monolayers were then infected with MAC 109, 2D6 or the complemented 2D6 mutant labeled with NHS-CF as described above using a MOI of 10. The cells were incubated for 4 h at 37°C for SP-D protein expression and 24 h for T-type Ca^++ ^alpha1I protein expression. The time points were chosen based on the expression results. The chambers were washed three times with sterile phosphate buffer saline (PBS) and treated with 200 μg/ml amikacin to kill extracellular bacteria. The cells were subsequently washed and allowed to air dry. Cells were then fixed with 2% paraformaldehyde for 1 h at room temperature, permeabilized in cold 0.1% Triton X-100 (J.T. Baker) and 0.1% sodium citrate for 20 min on ice. Next, the monolayers were washed with PBS and blocked with 2% BSA (BSA, Sigma) in PBS for 20 min at room temperature. The 2% BSA was replaced with 1 ml of specific primary antibody and allowed to incubate for 1 h. All the antibodies were prepared in 2% BSA in PBS to prevent non-specific binding. The cells were then washed three times with sterile PBS and re-incubated with the appropriate Texas-Red conjugated secondary antibody for an additional 1 h. Macrophages were washed three times with sterile PBS and allowed to air dry before adding Aqua-mount mounting media (Lerner laboratories, Pittsburgh, PA) and cover slips (Corning, Corning, NY). Cell preparations were visualized with a Leica DMLB microscope. The microscope was operated by Spot 3^rd ^Party Interface Software with a Photoshop CS version 8.0 on a Macintosh OS (version 4.0.9) based system.

### Immunoprecipitation and Western blot

The U937 cells were infected with *M. avium *wild-type or 2D6 mutant with MOI 1 cell:100 bacteria in 75 mc^2 ^flasks. After 30 min and 24 h following infections, monolayers were lysed and phagosomes were extracted as directed above. Equal amounts of phagosomal proteins were incubated with 10 μl of primary antibodies and 30 μl protein A/G Plus-agarose beads at 4°C overnight with continuous agitation. Next day, beads were washed three times with PBS, and the captured proteins were resolved on a 12% SDS-PAGE gel. Proteins were transferred into a nitrocellulose membrane and blocked overnight with Odyssey blocking buffer (Li-Cor) in TBS (Tris-buffered saline). The membranes were probed with EEA1, CREB-1, MARCO and α-tubulin antibodies (Santa Cruz Biotechnology) for 1 h and after, incubated with appropriate secondary antibodies (Li-Cor) in TBS for 1 h. Proteins were visualized by scanning of the membranes in the Odyssey Imager (Li-Cor, Lincoln, NE).

### Concentration of single elements in the phagosome

Human monocyte-derived macrophages were purified as previously described [17,28], seeded on 200-mesh Formvar-coated London finder gold grids (Electron Microscopy Sciences) and cultured in RPMI-1640 supplemented with 10% FBS. The monolayers were infected with mycobacteria (MOI 10) for 1 h and subsequently washed with PBS. The monolayers were maintained in culture for 1 h or 24 h, then fixed and prepared for x-ray microscopy, as previously reported [17,44], and the phagosome was obtained [17,44,45].

Elemental maps were extracted from x-ray fluorescence spectra, using the software package MAPS [47], and quantification was achieved by measuring x-ray fluorescence from NIST thin-film standards NBS 1832 and NBS 1833 (National Bureau of Standards, Gaithersburg, MD, USA), prior to, during, and after the experiments. Calibration curves and calculations were carried out as described [17,44,45]. Statistical analysis of observed elemental changes was performed by comparing the concentration of the respective elements using Student's-*t *test. A p < 0.05 was considered significant.

### Statistical analysis

Comparisons between control and experimental groups were submitted to statistical analysis to determine the significance. Statistical analysis of the means ± SD was determined by ANOVA. A p < 0.05 was considered significant. A DNA microarray was carried out three independent times, while the proteomic analysis of vacuole proteins was performed twice.

## Authors' contributions

SJ performed the proteomics, some of the DNA microarray, wrote the initial paper.

LD participated in all the steps of the paper.

DW, JM, IM, BL performed the x-ray microscopy.

YL participated in the microarray.

YY participated in the proteomic studies.

LEB directed the studies, helped in macrophage experiments, senior author.

All authors read and approved the final manuscript.
